# Supplementary material for: Family caregivers’ experience of caring for patients undergoing hemodialysis: A qualitative study at Muhimbili National Hospital in Dar es Salaam, Tanzania
Source: PLoS One. 2025 May 2;20(5):e0321732. doi: 10.1371/journal.pone.0321732 (PMC12047833; doi:10.1371/journal.pone.0321732)
Supplement: S2 Text — (DOCX) [file pone.0321732.s002.docx]

## A guide of In-Depth Interview for Family Caregivers’ Experience of Caring for Patients Undergoing Hemodialysis: A Qualitative Study at Muhimbili National Hospital in Dar es Salaam, Tanzania

Date of interview…………………………………..

Interview site………………………...

Interviewee No ………………………

Start time……………………………

End time………………………………

**A: Interviewee background**

Age of respondents----------------

Sex ------------------------------------------

Marital status---------------------------------------

Occupation -------------------------------------------

Level of education----------------------------------------

Family size--------------------------------------

Duration since the commencement of hemodialysis-------------------------

Relationship to the patient --------------------------------------------

**B: Family caregivers’ Experience**

Tell me about your life before you started caring for your loved one. How much did you know about kidney disease?

How did providing primary care for a loved one change the way you thought about your health? Can you give some examples of how caring for your loved one has altered you since you took on this role?

Please share with us your experience when caring for your patient

a). what is your positive experience?

b). What is your difficult experience?

What are the things that should be done to improve your care (experience)?

Thank you for your time in this interview; do you have anything else to add? If not, I appreciate it very much!

**END**
