# Supplementary material for: Family caregivers’ experience of caring for patients undergoing hemodialysis: A qualitative study at Muhimbili National Hospital in Dar es Salaam, Tanzania
Source: PLoS One. 2025 May 2;20(5):e0321732. doi: 10.1371/journal.pone.0321732 (PMC12047833; doi:10.1371/journal.pone.0321732)
Supplement: S3 Text — (DOCX) [file pone.0321732.s003.docx]

**Family Caregivers’ Experience of Caring for Patients Undergoing Hemodialysis: A Qualitative Study at Muhimbili National Hospital in Dar es Salaam, Tanzania**

**Table 1. Participants characteristics (n=14)**

| Gender | Male | 5 |
| --- | --- | --- |
|  | Female | 9 |
| Age(Median:36 years) | Below 30 | 5 |
|  | 30-49 | 7 |
|  | 50-69 | 2 |
| Level of education | Primary education | 5 |
|  | Secondary education | 6 |
|  | College and above | 3 |
| Duration of caregiving  (min: 6 months; max:  3 years; median: 17 months) | Less than a year | 1 |
|  | More than a year | 13 |
| Relationship to the  patient receiving  hemodialysis treatment | Parent | 4 |
|  | spouse | 5 |
|  | Sibling | 3 |
|  | Others | 2 |
| Employment status | Formal employment | 4 |
|  | Self-employed | 10 |

**Excerpts of Transcripts**

***Attitude of Healthcare Providers***

*Nurses attending to our patients should focus on their work because others are busy with other things...when you ask them something or want some clarification about the treatment, they don’t pay attention to you. (P11)*

*It depends...you may ask a nurse a question and she may answer you with a high tone, another may answer you with a normal tone, another will smile...so it is not uniform for all. It depends on the state of their mind at that time and day. (P3)*

*If you approach them in a good manner, they will respond to you well...but if you seem like challenging their profession...you will get problems and the interaction may not be smooth. (P10)*

*The nurses and doctors have good hearts and are very supportive. I asked one doctor for his phone number to call just in case my patient had a problem, and he gave it to me without hesitation. (P1)*

***Information Sharing and Decision-Making***

*I need to know a lot about the procedures to follow, the plan of care, and how to manage complications associated with the disease...I should not be left speculating like this. (P4)*

*I think due to the high number of patients they (healthcare providers) attend, they don’t have enough time for us to ask questions about our patients and procedures. We need dedicated time for meetings between doctors and us. (P7)*

***Access to Dialysis Treatment***

*It is saddening that I can only afford to bring my mother for a session once a week...sometimes I have to postpone treatment. Those with insurance can do so even thrice a day...you wish to bring your patient for treatment accordingly but you cannot afford it...the cost of treatment is very high. (P2)*

*The cost of treatment is not compatible with the income of an ordinary citizen...otherwise I would have brought my brother twice a week...but because of the high cost, sometimes eight days may pass...if the cost could be reduced to half...I could somehow afford. (P11)*

*I’m thankful that my patient has a health insurance plan that covers part of the dialysis treatment...so I’m not much stressed as I can manage other expenses. (P6)*

***Reliability of Dialysis Treatment***

*You find that the room has six machines and many patients are waiting...it takes a long time until your turn comes, by then your patient’s condition has worsened. (P3)*

*You may come here for the session and you are told there is no water (dialysate)...instead of doing the session at 10 am, you end up doing it at 4 pm... this is a problem. (P6)*

*I’m satisfied with the services provided; however, the problem is the shortage of staff...and sometimes nurses and doctors come late...instead of receiving treatment at 9 am, you get services at 11 am. (P12)*

***Emotional Impact***

*Caring for a kidney patient has taken a toll on my mental health due to the stress involved. Balancing the needs of an angry patient and putting on a happy face can be challenging and distressing. (P14)*

*Because you are involved with the patient for a long time, you become mentally sick too...you may find you speak to yourself, doing abnormal things...this means we also need some kind of counselling. (P4)*

*Taking care of a patient makes you unable to get more than four hours of sleep at night. In any case, you must awaken from the patient’s discomfort. You’ll most likely feel fatigued the next morning as a result of your lack of sleep. (P7)*

***Economic Impact***

*The process of caring for my mother has interfered with my normal life...the illness is the source of poverty. The family business has gone down because the money was just coming out to cover the treatment...but I don’t blame anyone for what is happening. (P12)*

*I used to think kidney failure is a disease for affluent people, but people with low life are now struck by it...Imagine you have a patient who spends a lot of money per month more than your salary...will you be able to afford treatment along with your family’s needs.... you can’t; it’s so difficult, this is what happening to me...! (P4)*

*Some close family members who are capable of helping are increasingly giving excuses for not being able to help our father, even being present for him. When you call them, they don’t answer your phone, because they know you are going to ask them for money...so you feel you are abandoned. (P5)*

*The situation has left my family in shambles...when you have financial difficulties bad things start to creep into your marriage, children start behaving badly especially girls...even boys start stealing things...all because of hunger in the house. (P9)*

***Religious Coping***

*I’m comforted by the word of God, you know everything is ordained by him, even this sickness. (P14)*

***Sharing Experiences***

*When you have this kind of burden you wish to have someone close to you who can understand your situation, when we meet with our fellows we talk together and exchange experiences...it helps a lot to navigate through same challenges. (P9)*
