# Supplementary material for: Family caregivers’ experience of caring for patients undergoing hemodialysis: A qualitative study at Muhimbili National Hospital in Dar es Salaam, Tanzania
Source: PLoS One. 2025 May 2;20(5):e0321732. doi: 10.1371/journal.pone.0321732 (PMC12047833; doi:10.1371/journal.pone.0321732)
Supplement: Attachment — Submitted filename: Review report - FCG - Dialysis - Tanzania .pdf [file pone.0321732.s004.pdf]

**Review report**

PONE-D-24-43236: Family caregivers' experience of caring for patients undergoing hemodialysis at Muhimbili National Hospital, Dar es Salaam, Tanzania

**Comments**

Very interesting study with relevant results pertinent to policy and action in low- and middle-income countries.

- Background: Need to improve this section by adding some global data and statistics from the global burden of diseases.
- Some areas need focused attention and further development – *p. 5 The main question was Tell me about your life when you started caring for your loved one in need of hemodialysis treatment. Unclear how one question generated the themes/subthemes- it will be helpful to attach data collection instrument – i.e. Interview Guide*.
- Clarity is needed on recruitment and sampling procedures; did the researchers just approach the FCG and talked to them about the study? At that point in time were the participants recruited into the study? Were there any benefits for the study participants?
- Was the sampling just purposive, how about convenience sampling?
- What was the study's inclusion and exclusion criteria?
- *P. 5 - All Interviews were conducted in Kiswahili language which is the national language of the participants.*-how did the translation to English affect data quality, what measures were put in place to ensure methodological rigor. How does this become study limitation noting that words in the local language and its translation into English does not always have its exact meaning in the English language.
- Need to number the themes and subthemes – the subthemes are quite not exhaustive – there is little information on system and structural support
- **P.**10 – main text and participant quotes need to be differentiated well – they appear lumped up. need to present quotes with due indents.
- The statements in between quotes need to be well presented, as best practice, these statements should be provided in between quotes and especially before transitioning to a theme or subthemes. Almost all the quotes ended without transition statements and new themes/subthemes were introduced.
- In relation to Employment status, was there any participant that was unemployed?
- Based on the study findings, the recommendations need to be quite clear – what are the implications for policy, practice and planning?
