# Supplementary material for: Family caregivers’ experience of caring for patients undergoing hemodialysis: A qualitative study at Muhimbili National Hospital in Dar es Salaam, Tanzania
Source: PLoS One. 2025 May 2;20(5):e0321732. doi: 10.1371/journal.pone.0321732 (PMC12047833; doi:10.1371/journal.pone.0321732)
Supplement: Attachment — Submitted filename: Response to Reviewers.docx [file pone.0321732.s006.docx]

**25/022025**

Editor-in-Chief

*PLOS ONE*

Dear Editor

**RE: Re-submission of a revised manuscript for publication**

I hereby re-submit a revised manuscript entitled “**Family Caregivers’ Experience of Caring for Patients Undergoing Hemodialysis: A Qualitative Study at Muhimbili National Hospital in Dar es Salaam, Tanzania”** for consideration for publication in *PLOS ONE* following addressing comments raised by the reviewers.

The response to the reviewer's comments sheet is attached below.

Best regards,

Menti Ndile

Lecturer-Department of Clinical Nursing,

Muhimbili University of Health and Allied Sciences.

**RESPONSE TO REVIEWERS' COMMENTS**

| **Reviewer 1** | | | |
| --- | --- | --- | --- |
|  | **Comment** | **Response** |  |
|  | The title should mention the type of study. | The title revised and it includes the type of study | Pg 1 |
|  | **Abstract** |  |  |
|  | An in-depth interview guide was used for data collection. It seems the word “guide” should be removed here | Thank you, the word “guide” has been removed as, suggested | Pg 2 |
|  | In the results section of the abstract, it should be specified how many individuals were interviewed, how many codes were extracted, and additional information should be provided to give the reader a clearer understanding. | Thank you. The number of interviewees has been included and additional information has been added to respective themes. | Pg 2 |
|  |  | Information about the number of codes extracted has been included in the main result section | Pg 8 |
|  | More keywords can be added, such as "caregiver burden," "care," etc. | Additional keywords have been added as suggested | Pg 2 |
|  | **Problem statement** |  |  |
|  | The knowledge gap has not been adequately addressed in the problem statement. Based on the current literature, what do we know and what do we not know? | The knowledge gap has been clarified to show the significance of the context of the study | Pg 3 |
|  | At the end of the problem statement, it should be explained in a paragraph why a qualitative research method is suitable for this study | The paragraph has been added to clarify the importance of qualitative study for this research | Pg 4 |
|  | **Methods** |  |  |
|  | It should be explicitly mentioned where the interviews were conducted. | Thank you. This is indicated in the Data collection and procedure section | Pg 5 |
|  | How did you ensure data saturation was achieved? Were any interviews repeated? | Thank you, the information has been added that saturation was observed through constant comparison of new and previous data from interviews- See Sample size and selection section | Pg 4 |
|  | During the interviews, was there anyone else present besides the interviewer and the participant? | In the data collection section, The sentence was added for clarity “Only the researcher and one participant were in the dedicated interview room” | Pg 5 |
|  | Did anyone withdraw from participating in the interview? | In the data collection section, The sentence was added for clarity “no FCG withdrew from the interview” | Pg 5 |
|  | How was coding agreement reached? | Thank you. The section on data analysis and Trustworthiness of the study shows agreement was reached through consensus agreement among co-authors | Pg 6 |
|  | What was your coding unit? In addition to interview content, were other data sources, such as field notes, used? | The main coding unit was interview content |  |
|  | A larger sample of interview questions should be provided. • Attach the interview guide. | The interview guide is attached as a supplementary file |  |
|  | To ensure the trustworthiness and credibility of research findings, the researcher had time to establish rapport with the participants and explain the purpose of the study; this established a trustful relationship between the researcher and individual participants. Participants were also informed that they were free to decline to participate, this ensured honesty in the voluntarily provided information." • It seems the rigor (trustworthiness) section needs further elaboration. Was peer-checking conducted? | Thank you. The mentioned statement has been removed. Collaborative analysis, peer checking and member checking were the strategies used to ensure rigor. An additional sentence has been added | Pg 6 |
|  | **Ethical consideration** |  |  |
|  | Was permission obtained to record the interviews? • Were participants allowed to refrain from answering any question they were uncomfortable with? Were they assured of confidentiality? These points should be mentioned in the ethical considerations. | Thank you. The section has been reorganised to include all suggested information | Pg 6 |
|  | **Results** |  |  |
|  | The number of initial codes obtained should be specified, and then, after merging similar ones, the number of final codes should be stated. | Thank you, the information about the number of codes has been added to the section | Pg |
|  | Each theme should have at least three sub-themes. | **Literature shows it is common to have at least 2 sub-themes as they can support the main theme (Braun & Clarke, 2006)** |  |
|  | The level of abstraction of the codes is very high. | Thank you. Some codes have been revised | Pg 8 and 9 |
|  | **Discussion/Conclusion** |  |  |
|  | It would be better to discuss the practical implications of the research findings at the end of the discussion or in the conclusion section. | Thank you. The paragraph on the implication of the study findings has been added | Pg 8 |
| **Reviewer 2** | | | |
|  | Background: Need to improve this section by adding some global data and statistics from the global burden of diseases. | Thank you. The section has been revised by adding some statistics about the burden of the disease | Pg 3 |
|  | Some areas need focused attention and further development – p. 5 The main question was Tell me about your life when you started caring for your loved one in need of hemodialysis treatment. Unclear how one question generated the themes/subthemes- it will be helpful to attach data collection instrument – i.e. Interview Guide. | Thank you for the comment. This was an opening question followed by other questions; this has been clarified. (Interview guide attached as supplementary file) | Pg 5 |
|  | - Clarity is needed on recruitment and sampling procedures; did the researchers just approach the FCG and talked to them about the study? At that point in time were the participants recruited into the study? | Thank you, in the data collection section, it explains that participants who agreed to participate were given appointments for interviews based on their convenience on subsequent visit to the renal unit(thrice per week). | Pg 5 |
|  | Were there any benefits for the study participants? | The only benefit explained in the consent process was that their information would help put strategies to improve the caring experiences of their patients | Pg 7 |
|  | - Was the sampling just purposive, how about convenience sampling? | The sampling was purposive although the selection of the study sight can be convenient |  |
|  | - What was the study’s inclusion and exclusion criteria? | Thank you. On the Sample Size Selection section it indicates that participants above 18 years with six months of caregiving roles and can express themselves well were selected. | Pg 4 |
|  | - P. 5 - All Interviews were conducted in Kiswahili language which is the national language of the participants.-how did the translation to English affect data quality, what measures were put in place to ensure methodological rigor. How does this become study limitation noting that words in the local language and its translation into English does not always have its exact meaning in the English language. | Thank you for the comment. English translation started at the level of coding-kiswahili codes were translated to English minimizing a risk of misinterpretation or loss of meaning during translation. The authors and external expert in qualitative analysis are fluent in both languages, this also minimized mistranslation. | Pg 6 |
|  |  | Information has been included as potential source of study limitation | Pg 19 |
|  | - Need to number the themes and subthemes – the subthemes are quite not exhaustive – there is little information on system and structural support | To reduce the mechanization of text and allow flow of the story, only the themes have been numbered while subthemes are italicized in the text | Pg 8-15 |
|  | - P.10 – main text and participant quotes need to be differentiated well – they appear lumped up. need to present quotes with due indents. | Thank you for the comment. The result section has been revised to make make differentiation | Pg 9-15 |
|  | - The statements in between quotes need to be well presented, as best practice, these statements should be provided in between quotes and especially before transitioning to a theme or subthemes. Almost all the quotes ended without transition statements and new themes/subthemes were introduced. | Thank you for a comment, The section has been revised and some statement added between quotes | Pg 9-15 |
|  | In relation to Employment status, was there any participant that was unemployed? | In Table 1. 10 participants were self-employed-doing their own income-generating activities without formal employment. | Pg 7 |
|  | - Based on the study findings, the recommendations need to be quite clear – what are the implications for policy, practice and planning? | Thank you. The paragraph about the implications of the study has been added | Pg 8 |
